# Meta-analysis reveals gender difference in the association of liver cancer incidence and excess BMI

**DOI:** 10.18632/oncotarget.20127

**Published:** 2017-08-10

**Authors:** Kun-Fang Yao, Ming Ma, Guo-Yong Ding, Zhan-Ming Li, Hui-Ling Chen, Bing Han, Qiang Chen, Xin-Quan Jiang, Li-Shun Wang

**Affiliations:** ^1^ Institute of Fudan-Minhang Academic Health System, Minhang Hospital, Fudan University, Shanghai, P.R. China; ^2^ School of Public Health Taishan Medical University, Shandong, P.R. China

**Keywords:** BMI, liver cancer, meta-analysis, incidence

## Abstract

Excess body weight has a positive association with risk of liver cancer, but the gender difference in the relationship between body mass index and liver cancer risk remains uncertainty. In this work, we performed meta-analysis for excess body weight and risk of liver cancer incidence to identify the gender difference. We searched the English-languages database and the Chinese literature databases to May 12, 2017. Overall, a total of 17 studies were included. Relative risks (RRs) with 95% confidence intervals was used to evaluate the strength of these associations. The RRs of liver cancer incidence for obese men and women were 2.04 (1.70–2.44) and 1.56 (1.37–1.78). The former one was significantly higher than the later one (P for interaction = 0.02). Notably, the RR of liver cancer incidence in non-Asian obese men was even higher than their counter part (2.31(1.85–2.91) vs. 1.56 (1.31–1.86), P for interaction = 0.01). Similar gender difference was observed in the dose-response curve. As example, at the point of BMI = 32 kg/m^2^, the RRs for men and women were 1.61 (1.45–1.79) and 1.41 (1.02–1.94) respectively. Findings from this meta-analysis indicate that obesity is associated with a higher risk of liver cancer incidence in men, especially in non-Asian men, which might partially contribute to the male dominance of liver cancer incidence.

## INTRODUCTION

Liver cancer is the sixth most prevalent neoplasm [1], an estimated 782,500 new liver cancer cases occurred worldwide during 2012 [2], whereas it has become the third most frequent cause of death from cancer [3]. Chronic Hepatitis B or C viral infection, alcohol consumption, cigarette smoking, non-alcoholic steatohepatitis and aflatoxin exposure have been identified as key risk factors for this cancer [4–7].

One universal epidemiologic characteristic of liver cancer is the prominent male dominance [8]. It is the fifth most common cancer in men and the ninth in women [9, 10]. Stimulatory effects of androgen and the protective effects of estrogen has been suggested as the cause [11]. Potential sexual dimorphism in liver cancer is possibly caused by the differential recruitment of Foxa-1/2 transcription factors and the corresponding androgen and estrogen receptors [12]. However, the causes for the gender difference still need further investigation.

Obesity is growing globally, the worldwide prevalence of obesity has doubled from 1980 to 2008 [13]. There were multiple evidences suggesting that excess body weight increases liver cancer risk [14–17]. As an example, patients with a history of obesity have a 2.47-fold higher liver cancer risk [18]. However, the gender difference for the effect of body mass index (BMI) on liver cancer incidence is uncertain. Thus, this meta-analysis was conducted to quantitatively and precisely evaluate the gender difference of this relevance.

## RESULTS

### Literature search and study characteristics

**Figure 1:**
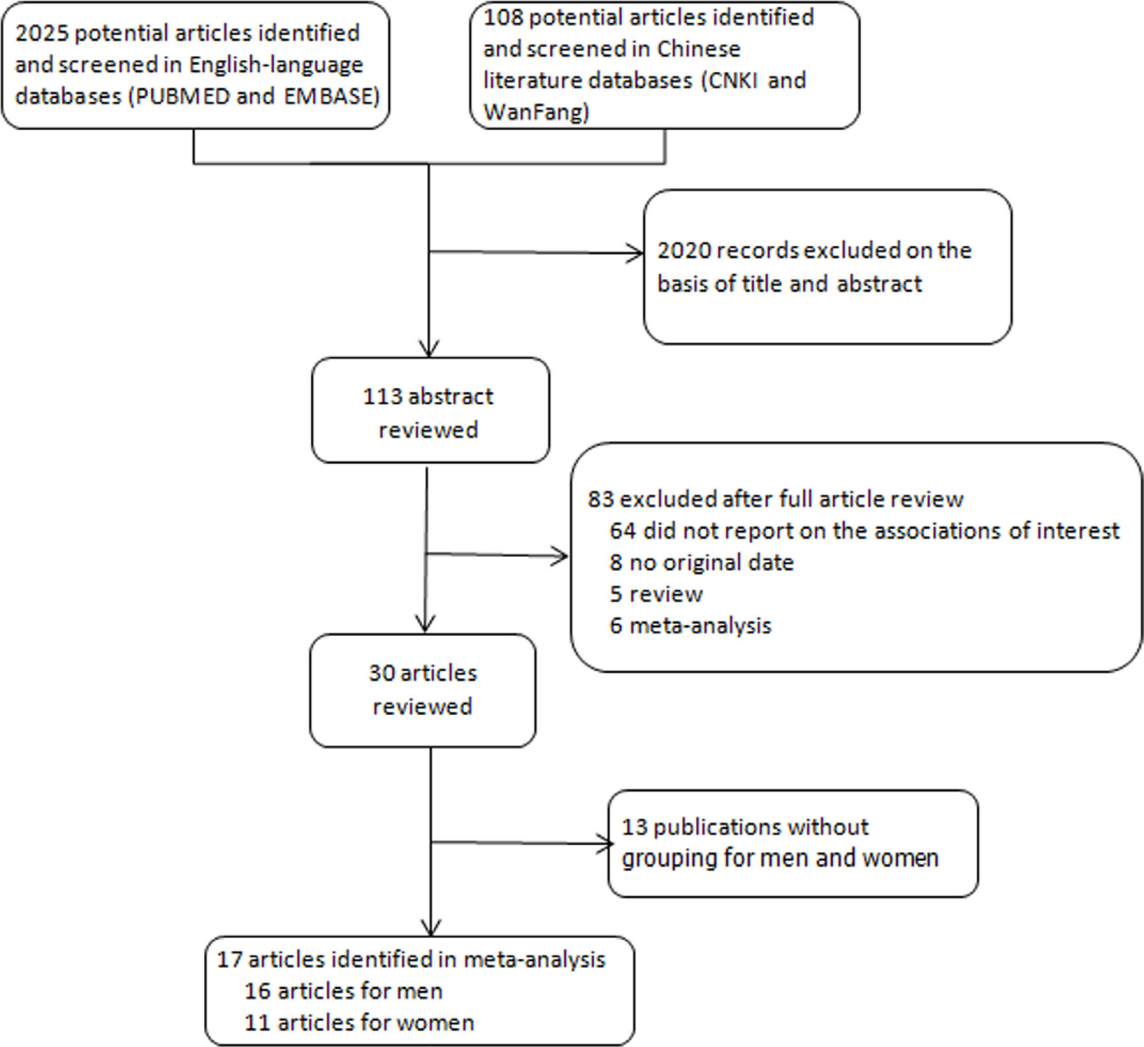
Flow diagram of the study selection in this meta-analysis

As shown in Figure 1, for BMI and liver cancer incidence, we identified 17 relevant articles [19–35], including 13 cohort studies [19, 20, 23–28, 30, 31, 33–35] and 4 case-control studies [21, 22, 29, 32], with a total of 18225 cases. All studies reported outcomes of both sex. The quality score of studies ranged from 6 stars to 9 stars (Supplementary Table 1) according to the 9-star Newcastal-Ottawa Scale [30]. Characteristics of studies included in the meta-analysis were presented in Table 1.

**Table 1:** Characteristics of included studies

Author, year, country	Study type	Age ranges	Follow up, year	study size no.	No of cases	Assessment method of weight/height	BMI (Kg/m2)	RR		Adjustment factors
								men	women	
Campbell el al, 2016, US	cohort	58.2 average	12	1.57 million	M:1463 W:386	self–reported	< 18.5 18.5–24 25–29 ≥ 30	1.47 (0.73–2.96) 1.00 (reference) 1.24 (1.08–1.42) 1.88 (1.61–2.18)	1.35 (0.74–2.48) 1.00 (reference) 1.03 (0.85–1.24) 1.56 (1.27–1.90)	Age, sex, study, alcohol, cigarette smoking, race, and diabetes
Guo et al, 2014 northern China	cohort	51.07 ± 13.54, average	4.28	M: 106630	M: 127	Measured	<18.5 18.5–24 24–28 ≥28	3.00 (1.36–6.65) 1.00 (reference) 0.83 (0.54–1.27) 1.08 (0.60–1.92)	NA	Age, education, smoking, alcohol, HBsAg
Hagstrom et al, 2017 Sweden	cohort	17–19	28.5	M:1220261	M:251	Measured	< 18.5 18.5–< 22.5 22.5–< 25 25–< 30 ≥ 30	1.12 (0.74–1.68) 1.00 (reference) 1.28 (0.91–1.80) 1.57 (1.01–2.45) 3.59 (1.85–6.99)	NA	Age,year of birth, location of conscription, education, parental socioeconomic status, scores on intelligence test, cardiovascular capacity and muscular strength tests, systolic and diastolic blood pressures
Inoue et al, 2009, Japan	cohort	40–69, range	10.2	M:9548 W:18176	M:27 W:18	Measured	overweight	2.18 (1.33–3.58)	1.95 (1.03–3.69)	Age, sex, smoking, alcohol, HBV, HCV, coffee intake
Jee et al, 2008 korea	cohort	30–95, range	10.8	M: 770556 W: 443273	M: 8759 W: 1761	Measured	< 20.0 20.0–22.9 23.0–24.9 25.0–29.9 ≥ 30.0	0.90 (0.81–1.00) 0.97 (0.90–1.04) 1.00 (reference) 1.04 (0.96–1.13) 1.63 (1.27–2.10	0.85 (0.67–1.06) 0.76 (0.64–0.91) 1.00 (reference) 1.14 (1.97–1.35) 1.39 (1.00–1.94)	Age, smoking
Kuriyama et al, 2005, Japan	cohort	≥ 40, range	7.6	M: 12485 W: 15054	M: 69 W: 31	Self–report	18.5–24.9 25.0–27.4 27.5–29.9 ≥ 30.0	1.00 (reference) 0.80 (0.40–1.63) 1.14 (0.46–2.87) —	1.00 (reference) 1.30 (0.54–3.16) 0.91 (0.30–2.80) —	Age, smoking, meat, vegetables, alcohol intake, bean–paste soup, type of health insurance
Liu et al, 2016 chinese	cohort	40–70, range	15.1	W: 68253	W: 165	Measured	18.5–22.9 ≥ 30	NA	1.00 (reference) 1.93 (1.14–3.27)	Age, education, alcohol, smoking, family history of cancer, menopausal status
Moller etal, 1994, Danish	cohort	all	4.8	M:14531 W:29434	M:22 W:36	Discharge diagnosis ofobesity	obesity	1.9 (1.2–2.9)	1.9 (1.4–2.7)	Age
Oh et al, 2005, korea	cohort	≥20, range	10	M: 781283	M: 3347	Measured	< 18.5 18.5–22.9 23.0–24.9 25.0–26.9 27.0–29.9 ≥ 30.0	0.84 (0.63–1.10) 1.00 (reference) 1.04 (0.96–1.13) 1.04 (0.94–1.14) 1.07 (0.93–1.23) 1.56 (1.15–2.12)	NA	Age, area of residence, family history of cancer, smoking, exercise, alcohol
Pan et al, 2004, Canada	case–control	20–76, range		M:14047 W:12014	M:225 W:84	Discharge diagnosis ofobesity	< 25 25 – < −30 ≥ 30	1.00 (reference) 0.99 (0.72–1.38) 1.30 (0.85–1.97)	1.00 (reference) 0.61 (0.35–1.07) 0.94 (0.48–1.84)	Age, education, smoking, alcohol, total caloric intake, vegetable intake, dietary fiber intake, physical activity
Petrick et al, 2016, Northern California	case–control	NA		M: 2409 W: 1217	M: 238 W: 118	Self–report	< 18.5 18.5–< 25 25–< 30 ≥ 30	2.19 (0.72–6.61) 1.00 (reference) 1.31 (0.97–1.78) 2.68 (1.73–4.16)	0.77 (0.18–3.33) 1.00 (reference) 1.41 (0.90–2.23) 2.00 (1.14–3.52)	Birth cohort, race/ethnicity, sex, alcohol, smoking status, education
Rapp et al, 2005, Austrian	cohort	35–54, range	9.9	M: 67447	M: 57	Measured	18.5–24.9 25–29.9 30–34.9	1.00 (reference) 1.32 (0.73–2.37) 1.67 (0.75–3.72)	NA	Age, smoking, occupational group
Samanic et al,2006, sweden	cohort	34.3, average	19	M: 362552	M: 297	Measured	18.5–24.9 25.0–29.9 ≥30	1.00 (reference) 1.29 (1.00–1.68) 3.62 (2.62–5.00)	NA	Age, smoking
Setiawan et al,2016, Hawaii and California	cohort	45–75, range	16.6	M: 58937 W: 90402	M: 339 W: 143	Self–report	< 25 25–< 30 ≥ 30	1.00 (reference) 1.50 (1.16–1.95) 1.82 (1.31–2.52)	1.00 (reference) 0.98 (0.65–1.48) 1.32 (0.83–2.11)	Age, race/ethnicity, education, diabetes, smoking status, alcohol intake
Trichopoulosetal, 2011, Europe or NorthAmerica	case–control	25–70, range		M: 239 W: 105	M: 80 W: 35	Measured	<30 ≥30.0	1.00 (reference) 3.66 (1.46–9.14)	1.00 (reference) 0.57 (0.15–2.12)	Age, education, smoking, coffee intake, HBV, HCV, ethanol intake
Wolk et al,2001, Sweden	cohort	≥18, range	10.3	M:8165 W:19964	M:15 W:13	Discharge diagnosis ofobesity	obesity	3.6 (2.0–6.0)	1.7 (0.9–2.9)	Age, calendar year
Yu et al,2001, Taiwan	case–control	≥30, range		M: 4841	M: 119	Self–report	16.7–22.0 22.1–24.5 24.6–32.0	1.00 (reference) 1.52 (0.81–2.87) 1.98 (1.05–3.74)	NA	Age, the time of blood draw, ethnicity, education, smoking, alcohol, history of chronic liver disease

### Association between BMI and liver cancer incidence

Compared to the reference category (normal weight), a positive association was observed between high BMI and liver cancer incidence (overweight: 1.16 (1.08–1.25); obesity: 1.83 (1.60–2.09)) (Supplementary Figure 1).

### Subgroup analysis for the association between BMI and liver cancer incidence

**Figure 2:**
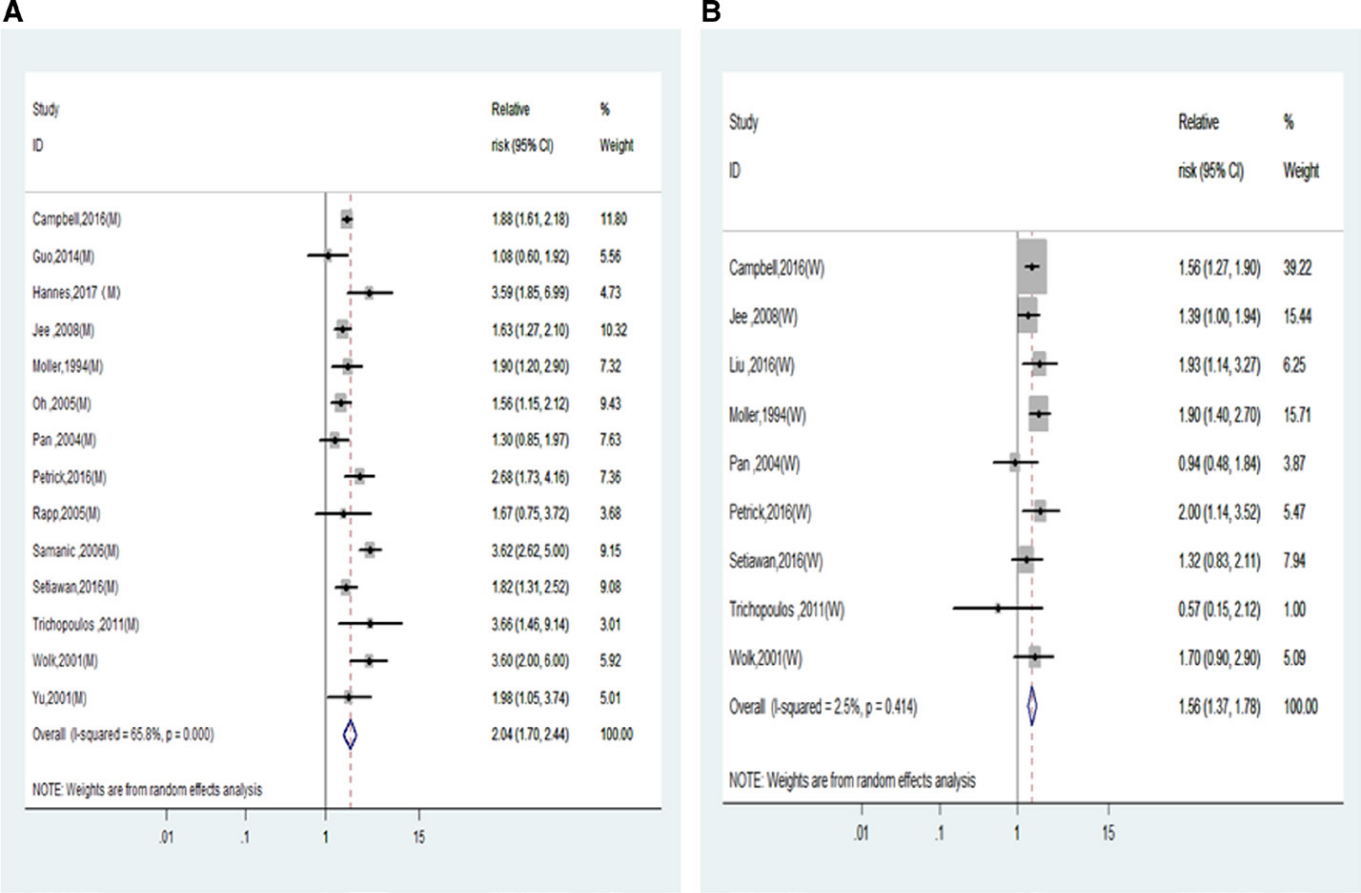
Relative risks of liver cancer incidence in obesity of overall population (**A**) Forest plots of liver cancer incidence RR in obese men; (**B**) Forest plots of liver cancer incidence RR in obese women. RR, relative risk; BMI, body mass index.

Subgroup analysis by sex were conducted to further examine the association of BMI and liver cancer incidence. As shown in Figure 2 and Table 2, the RRs of liver cancer incidence for men and women in the category of obesity were 2.04 (1.70–2.44) and 1.56 (1.37–1.78). Interaction analysis was conducted to compare the RRs between men and women and the P for interaction is 0.02, which indicated that the risk of liver cancer incidence was significantly higher in men than in women. Instead, the interaction analysis indicated there was no significant difference between the RRs of overweight men and women (1.18(1.01,1.30) vs. 1.11(1.00,1.24), *P* for interaction = 0.47) (Table 2).

**Table 2:** Subgroup analyses of BMI and liver cancer incidence

	Overweight		Heterogeneity		interaction	Obesity		Heterogeneity		interaction
	No. of studies	RR (95 CI%)	P	*I*^2^ (%)	P	No. of studies	RR (95 CI%)	P	*I*^2^	P
Over all Incidence	13	1.16 (1.08, 1.25)	0.016	45.0		15	1.83 (1.60, 2.09)	0	59	
Sex										
Men	13	1.18 (1.01, 1.30)	0.004	58.7		14	2.04 (1.70, 2.44)	0	65.8	
Women	7	1.11 (1.00, 1.24)	0.500	0	0.47	9	1.56 (1.37, 1.78)	0.414	2.5	0.02
Study location										
Non-Asia	7	1.21 (1.11, 1.33)	0.365	8.3		10	1.95 (1.64, 2.31)	0	64.6	
Asia	6	1.10 (1.00, 1.22)	0.050	48, 5	0.16	5	1.56 (1.34, 1.81)	0.656	0	0.05
Non-Asia										
Non-Asia (M)	7	1.28 (1.16, 1.40)	0.559	0		10	2.31 (1.85, 2.91)	0.001	67.9	
Non-Asia (W)	4	1.06 (0.90, 1.23)	0.600	0	0.04	7	1.56 (1.31, 1.86)	0.312	15.5	0.01
Asia										
Asia (M)	6	1.07 (0.96, 1.20)	0.049	55.1		4	1.57 (1.32, 1.87)	0.534	0	
Asia (W)	3	1.23 (0.95, 1.59)	0.275	22.5	0.33	2	1.53 (1.14, 2.06)	0.301	6.4	0.88
Study location										
Sweden	2	1.36 (1.08, 1.70)	0.453	0		3	3.07 (2.19, 4.32)	0.148	43.8	
Non-Sweden	11	1.16 (1.05, 1.28)	0.006	59.6	0.21	12	1.60 (1.52, 1.85)	1	0	0
Study design										
Cohort	10	1.16 (1.06, 1.26)	0.007	53.8		11	1.85 (1.60, 2.14)	0.001	61.7	
Case-control	3	1.20 (1.00, 1.44)	0.526	0	0.74	4	1.72 (1.19, 2.49)	0.025	58.4	0.72
Disease type										
Liver cancer	10	1.13 (1.05, 1.21)	0.054	39, 3		11	1.81 (1.56, 2.09)	< 0.001	62.4	
HCC	3	1.38 (1.12, 1.70)	0.325	13.5	0.08	4	1.93 (1.33, 2.80)	0.146	41.3	0.75
Duration of follow-up (cohort studies only )										
≥ 10	7	1.20 (1.09, 1.33)	0.001	67.4		6	1.84 (1.54, 2.19)	0	66.9	
< 10	3	0.98 (0.75, 1.28)	0.621	0	0.16	4	1.88 (1.42, 2.50)	0.107	44.8	0.90
Study size										
≥ 30000	7	1.11 (1.04, 1.20)	0.059	45.1		8	1.73 (1.47, 2.03)	0.002	64.9	
< 30000	6	1.32 (1.11, 1.57)	0.203	26.1	0.07	7	1.99 (1.57, 2.51)	0.015	53.2	0.34
Adjustment factors										
Smoking										
yes	12	1.15 (1.07, 1.24)	0.022	43.7		11	1.72 (1.47, 2.01)	<0.001	62.6	
no	1	1.57 (1.01, 2.45)			0.17	4	2.20 (1.73, 2.81)	0.2	31.5	0.09
Alcohol										
yes	9	1.18 (1.06, 1.31)	0.013	50.5		9	1.67 (1.45, 3.51)	0.072	38.3	
no	4	1.14 (1.01, 1.29)	0.186	35.3	0.68	6	2.14 (1.64, 2.78)	0	71.5	0.35

The prevalence of obesity is markedly different between in Western countries and in Asian countries [36]. Thus we compared the RRs between in non-Asian countries and Asian countries. In the subgroup analysis by study location, the RRs for liver cancer incidence in non-Asian were 1.21 (1.11, 1.33) and 1.95 (1.64,2.31) respectively for overweight and obesity (Supplementary Figure 2 and Table 2), while the corresponding RRs for Asian were 1.10 (1.00, 1.22) and 1.56 (1.34, 1.81). The RR of liver cancer incidence was slightly higher in non-Asian obesity than Asian obesity (P for interaction = 0.05) (Table 2). However, the RR was significantly higher in Sweden studies than that in non-Sweden studies (3.07 (2.19–4.32) vs. 1.68 (1.52–1.85), *P* for interaction = 0.00, Table 2).

**Figure 3:**
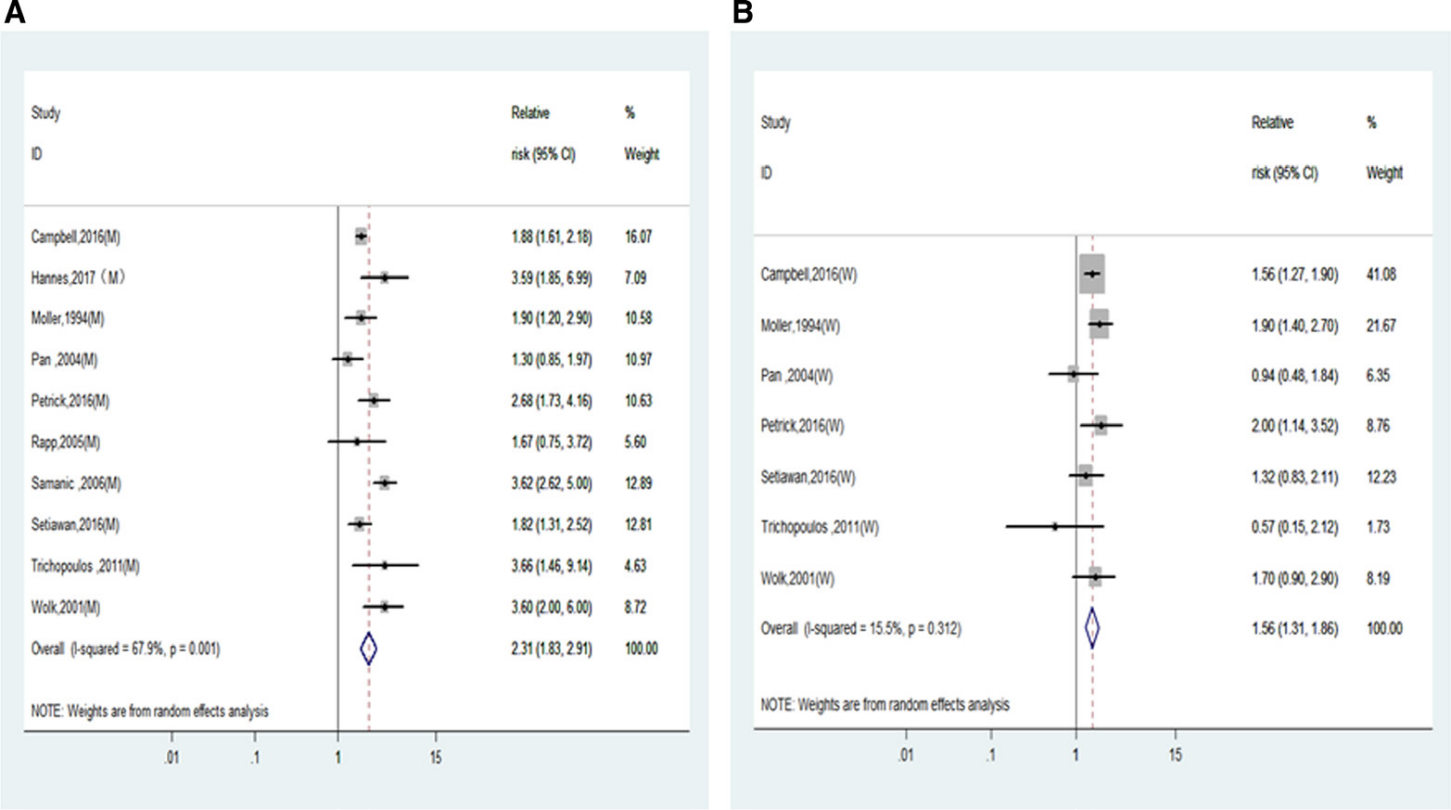
Relative risks of liver cancer incidence in obesity of non-Asian population (**A**) Forest plots of liver cancer incidence RR in obese men; (**B**) Forest plots of liver cancer incidence RR in obese women. RR, relative risk; BMI, body mass index.

Furthermore, the RRs of liver cancer incidence for men and women were calculated and compared in non-Asia countries and Asia countries separately. As shown in Figure 3 and Table 2, the RRs of liver cancer incidence were significantly higher in men than in women in non-Asian studies (2.31 (1.85–2.91) vs. 1.56 (1.31–1.86), P for interaction = 0.01) but not in Asian men and women (1.57 (1.32–1.87) vs. 1.53 (1.14–2.06), *P* for interaction = 0.88).

There were no differences between HCC and overall liver cancer, study design, disease type, duration of follow-up, study size, adjustment factors (Table 2).

### Dose-response meta-analysis

**Figure 4:**
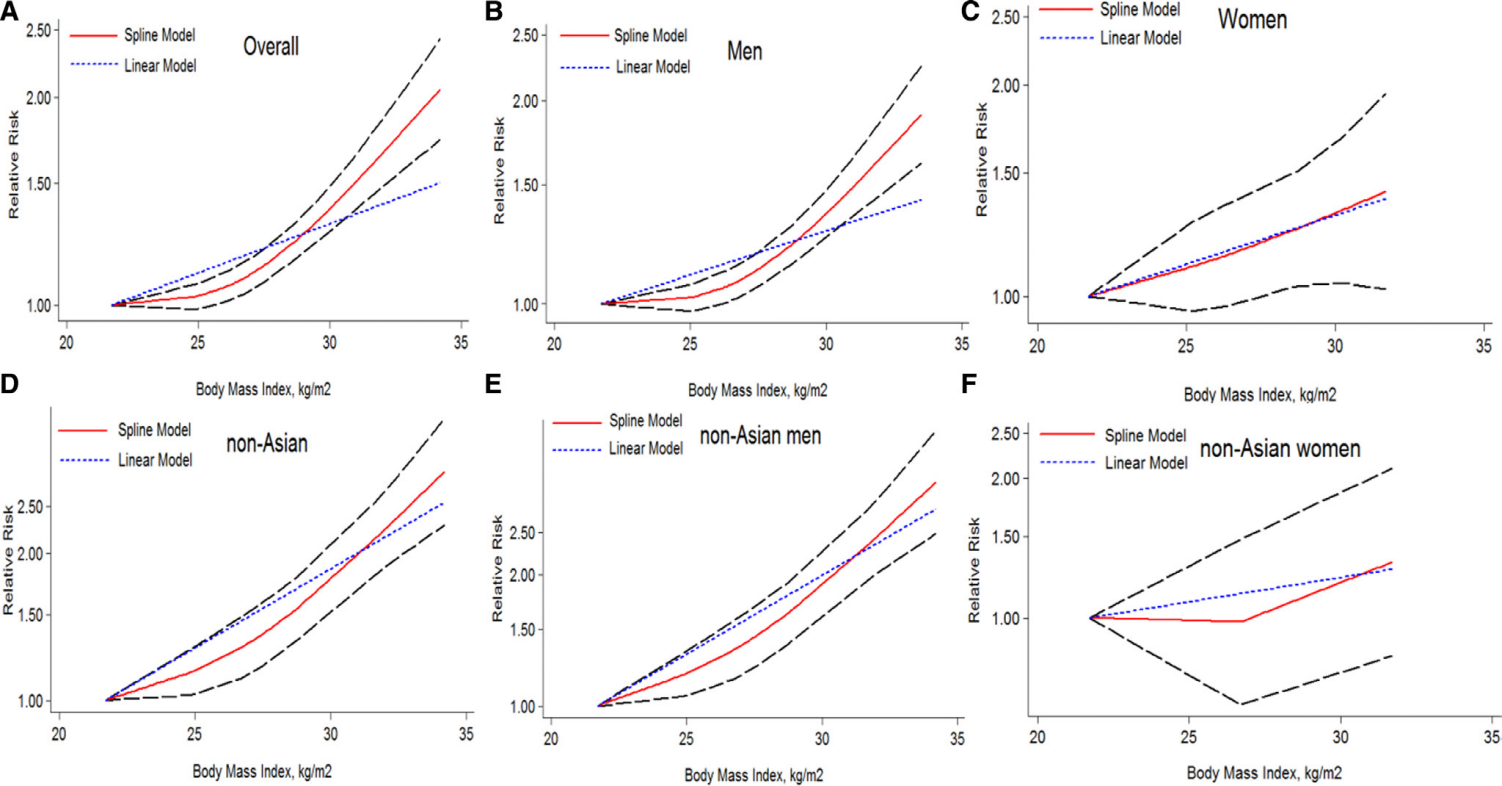
The dose-response analysis of BMI and liver cancer incidence risk The short dash line represents the linear relationship (per 1 kg/m^2^ increment). The solid line and the long dash line represent the estimated RR and its 95% CI respectively: (**A**) overall (1.04 (1.02–1.07) *p* = 0.000); (**B**) men (1.04(1.01–1.07) *p* = 0.000); (**C**) women (1.03 (1.01–1.06) *p* = 0.018); (**D**) non-Asian (1.07 (1.04–1.10) *p* = 0.000); (**E**) non-Asian men (1.08 (1.06–1.11) *p* = 0.000); (**F**) non-Asian women(1.02 (0.98–1.07) *p* = 0.301). RR, relative risk; CI, confidence interval; BMI: body mass index.

Furthermore, we assessed the dose-response relationship between BMI and liver cancer incidence with 8 studies [23–25, 27, 30, 33–35]. And we found a nonlinear dose-response (*P* = 0.000) relationship between BMI and the risk of liver cancer incidence. This meta-analysis showed an increased liver cancer incidence of 4% for each 1 kg/m^2^ increment in BMI as shown in Figure 4A. When adjusted for sex, the risk of liver cancer incidence was increased faster in men (Figure 4B) than in women (Figure 4C). As example, at the point of BMI = 32 kg/m^2^, the RRs for men and women were 1.61 (1.45–1.79) and 1.41 (1.02–1.94) respectively (Figure 4B and 4C). Similarly, nonlinear dose-response (*P* = 0.000) meta-analysis was found in non-Asian studies, which showed an increased risk of 7% for each 1 kg/m2 increment in BMI as shown in Figure 4D. The risk increment was more significant in men (Figure 4E) than in women (Figure 4F). As example, the corresponding RR at the point of BMI = 32 kg/m2 for men and women were 2.34 (1.94–2.82) vs. 1.32 (0.83–2,10) (Figure 4E and 4F). Notably, there was only one study available for the does-response meta-analysis in non-Asian women (Figure 4F), this plot need further verification.

### Meta-regression analysis and sensitivity analysis

Heterogeneity were detected in overweight and obesity men (Table 2). The meta-regression analysis was performed to investigate whether the association between BMI and liver cancer risk was modified by study location, publication year, study size, etc. We found that the study location can explain heterogeneity in overweight group (Non-Asia: *I*^2^ = 8.3%, Asia: *I*^2^ = 48.5%). Study location of Sweden could explain heterogeneity in obesity group (Sweden: *I*^2^ = 43.8%, Non-Sweden: *I*^2^ = 0%). The heterogeneities decreased significantly when these Sweden studies were stratified in the group (Table 2) verifying these heterogeneities were contributed by these Sweden studies.

### Sensitivity analysis

In a influence analysis in which one study at a time was removed and the rest was analyzed, the summary RR were not materially altered between BMI and liver cancer incidence risk (Supplementary Figure 3). Similarly, the summary RR were not materially altered in the overall obesity men and women (Supplementary Figure 4), as well as non-Asian obesity men and women (Supplementary Figure 5), supporting the robustness of our results.

### Publication bias

**Figure 5:**
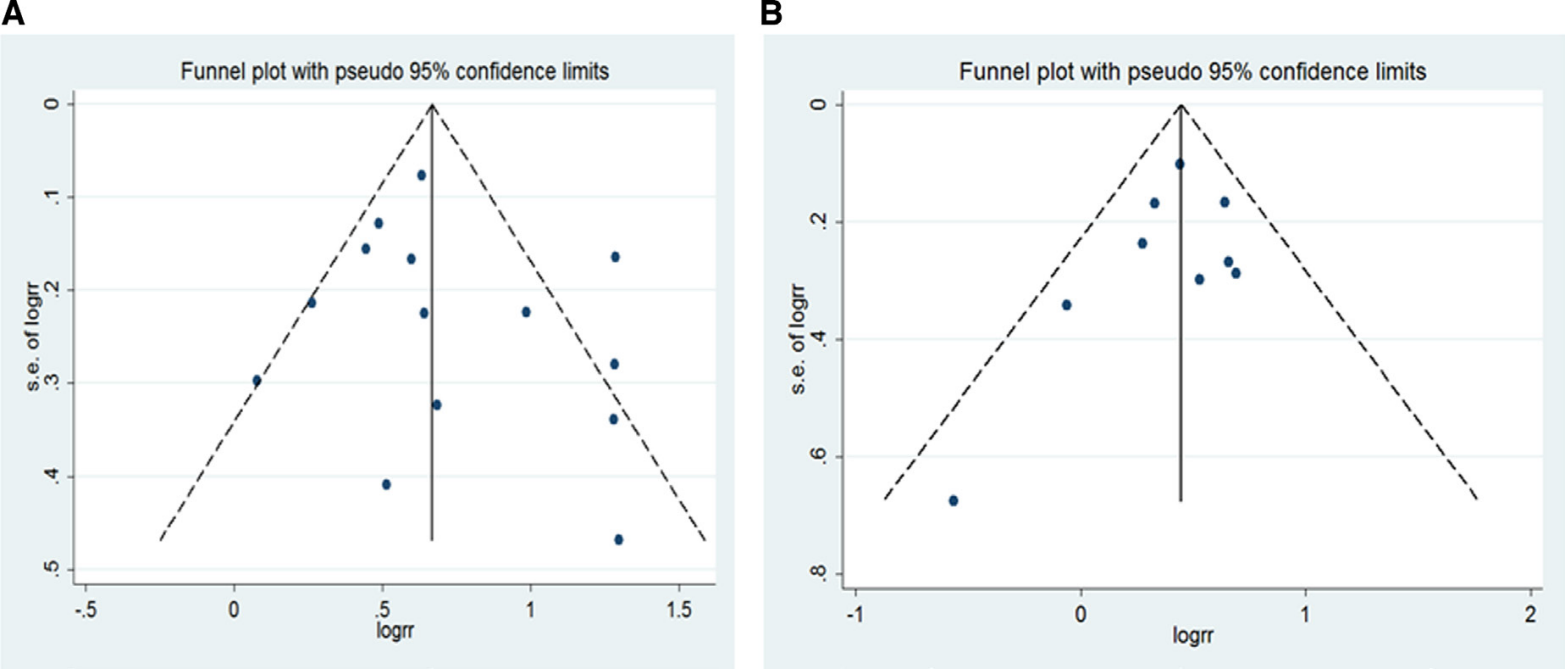
Funnel plot for all studies included in the meta-analysis of BMI and liver cancer incidence risk (**A**) obese men (*p* = 0.429 by Egger's test); (**B**) obese women (*p* = 0.370 by Egger's test).

**Figure 6:**
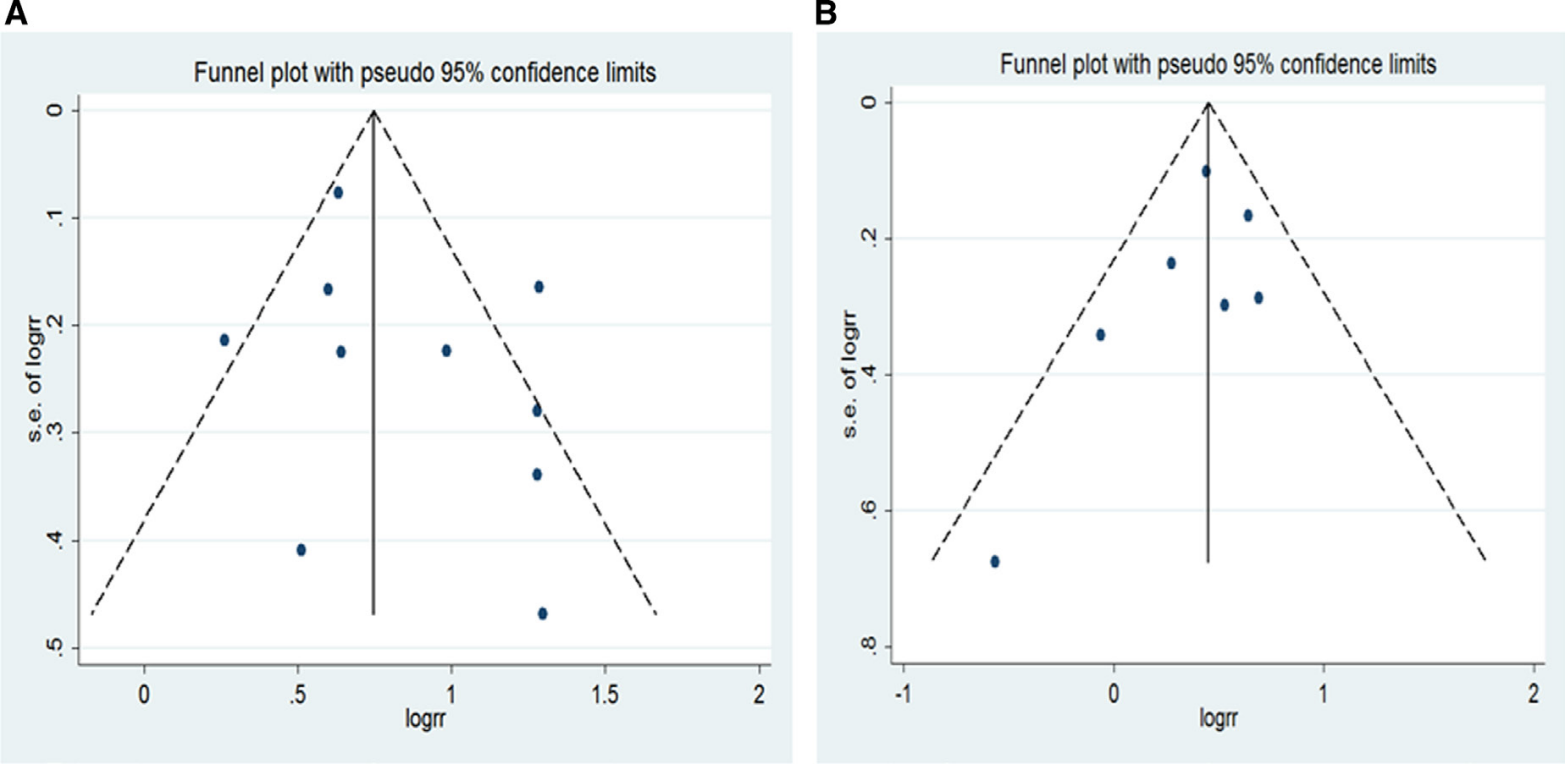
Funnel plot for non-Asian studies included in the meta-analysis of obesity and liver cancer incidence risk (**A**) obese men (*p* = 0.266 by Egger's test); (**B**) obese women (*p* = 0.294 by Egger's test).

For BMI and liver cancer incidence in the overweight group, the Egger's test showed the possibility of publication bias for the analysis (*p* = 0.022) (Supplementary Figure 6A). However, when the“trim and fill”approach was performed, data was unchanged, suggesting that the effect of publication bias could be negligible. No evidence for publication bias was indicated by Egger's regression test in the literature on BMI and liver cancer incidence in overall obesity group (*P* = 0.900) (Supplementary Figure 6B), as well as non-Asian overweight (*p* = 0.877) and obesity (*p* = 0.794) (Supplementary Figure 7). Similarly, no publication bias addressing the effect of overall obesity (men: *p* = 0.429;women: *p* = 0.370) (Figure 5) as well as non-Asian obesity (men: *p* = 0.266; women: *p* = 0.294) (Figure 6).

### Trial sequential analysis

**Figure 7:**
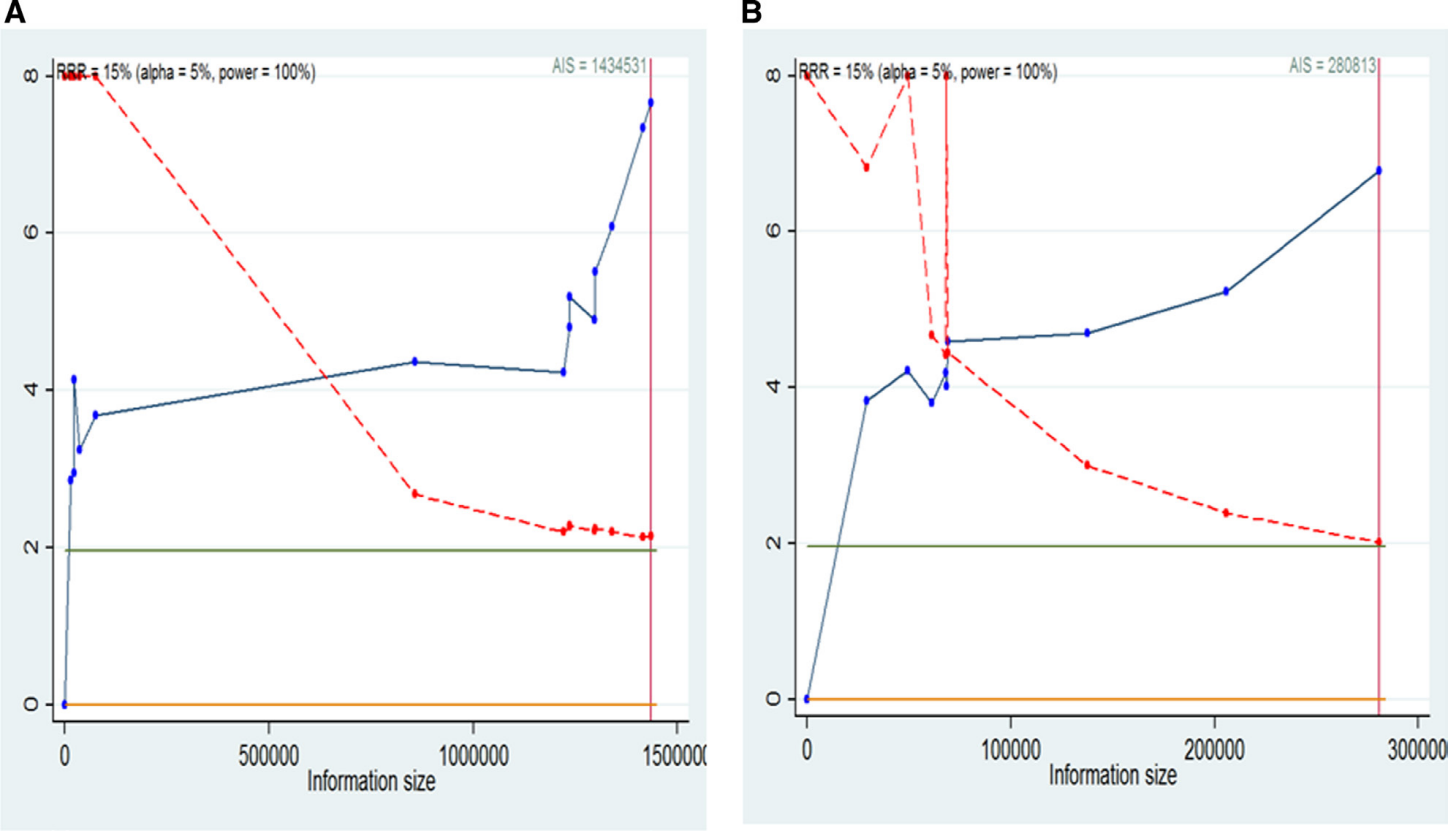
Trial sequential analysis for BMI and liver cancer incidence in overall men and women (**A**) Trial sequential analysis of men. The AIS = 1434531,α = 0.05, power = 100%; (**B**) Trial sequential analysis of women. The AIS = 280813,α = 0.05, power = 100%. A full blue cumulative Z-curve did cross the conventional boundary for benefit and did cross the AIS boundary. RRR, relative risk reduction; AIS, accrued information size.

**Figure 8:**
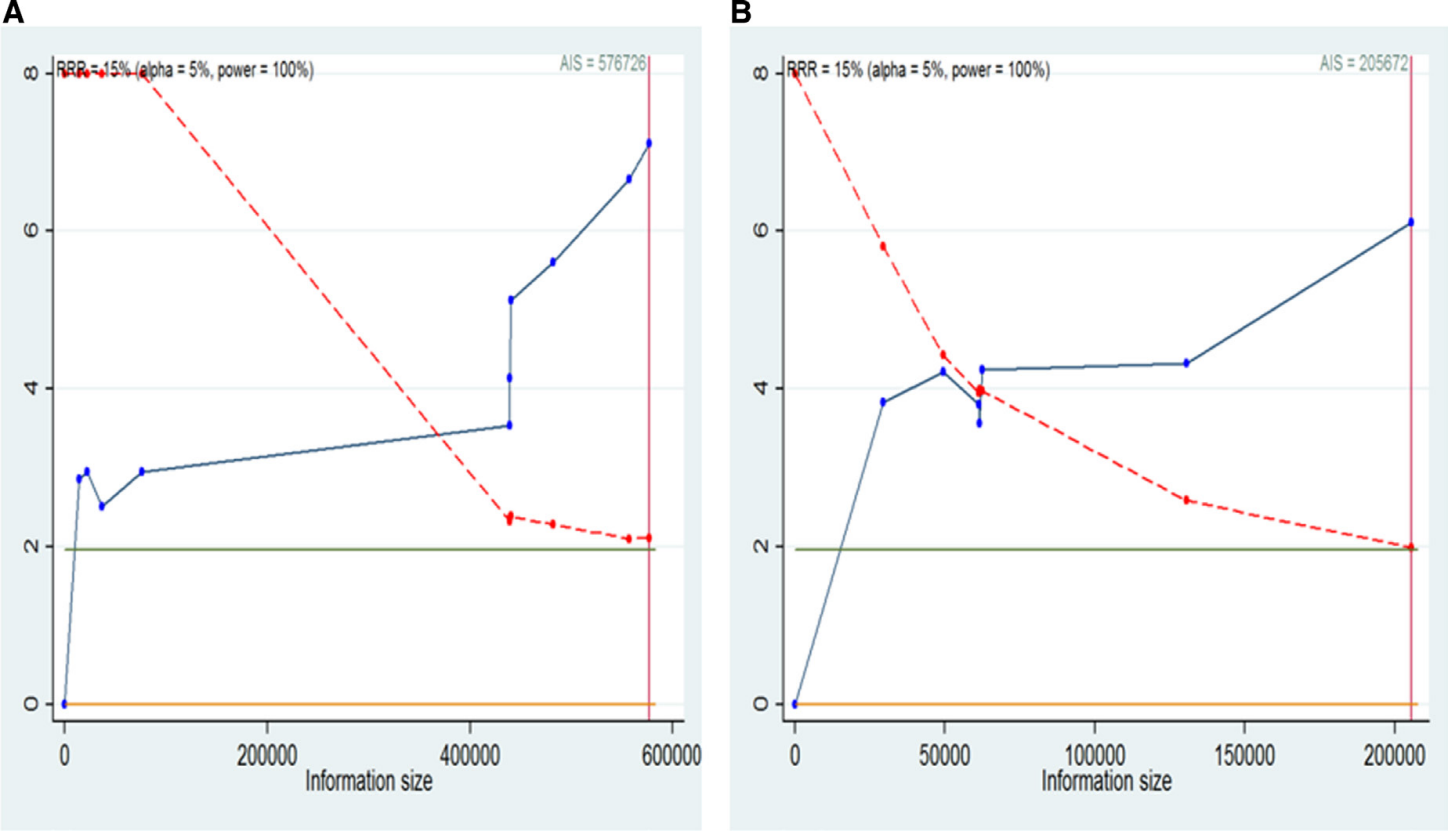
Trial sequential analysis for BMI and liver cancer incidence in non-Asian men and women (**A**) Trial sequential analysis of men. The AIS = 576726, α = 0.05, power = 100%; (**B**) Trial sequential analysis of women. The AIS = 205672,α = 0.05, power = 100%. A full blue cumulative Z-curve did cross the conventional boundary for benefit and did cross the AIS boundary. RRR, relative risk reduction; AIS, accrued information size.

We will conduct a formal trial sequential analysis (TSA) [37] by using the optimal event size to help to construct sequential monitoring boundaries for our meta-analysis. TSA analysis were conducted for the studies in overall overweight (Supplementary Figure 8A), overall obesity (Supplementary Figure 8B), non-Asian overweight (Supplementary Figure 9A), non-Asian obesity (Supplementary Figure 9B), overall obesity men (Figure 7A), overall obesity women (Figure 7B), non-Asian obesity men (Figure 8A) and non-Asian obesity women (Figure 8B). These cumulative Z-curves did cross the conventional boundaries for benefit and the accrued information size (AIS) boundaries. These results indicated that conclusive evidences were established and that further trials were not required.

### Power analysis

Power analysis were conducted and the power value (Table 3) was 97.17%, 100%, 99.6%, 100%, 100%, 99.99%, 99.99% and 99.99% for the studies in overall overweight (RR = 1.16), overall obesity (RR = 1.83), non-Asian overweight (RR = 1.21), non-Asian obesity (RR = 1.95), overall obesity men (RR = 2.04), overall obesity women (RR = 1.56), non-Asian obesity men (RR = 2.31) and non-Asian obesity women (RR = 1.56) respectively. These power values suggested the quality of evidence were determined to be high.

**Table 3:** Power value of the evidence in this meta-analysis

Type	No. of studies	Overweight (Power %)	No. of studies	Obesity (Power %)
Overall	13	97.17	15	100
Men	-	-	14	100
Women	-	-	9	99.99
Non-Asian	7	99.6	10	100
Non-Asian(men)	-	-	10	99.99
Non-Asian(women)	-	-	7	99.99

## DISCUSSION

Men develop liver cancer more often than women. However, the causes for the gender difference still need to be explored. In this meta-analysis, the liver cancer incidence risk in obesity was found to be higher in non-Asian men than in non-Asian women. Further dose-response analysis showed that a tendency towards higher liver cancer incidence was also seen in men.

The association between BMI of liver cancer incidence is well documented [15–17]. Instead, we tested the gender difference of the BMI effect on liver cancer risks. The previous meta-analysis [14, 16, 38] tried to test the gender difference for the effect of BMI on liver cancer risks with the combination risk data of liver cancer incidence and mortality. However, liver cancer incidence and mortality are defined differently and the combination in these studies make the association analysis vague.

In our meta-analysis, 17 studies were included and we applied TSA to reduce the risk of type I error and testify whether the evidence of our results was reliable. These results indicated that conclusive evidence was established and that further trials were not required. In addition, the power analysis suggests that the meta-analysis have high statistical power. Based on these solid risk data, interaction statistics between men and women have been further analyzed. In addition, a dose-response meta-analysis is firstly conducted for BMI and liver cancer incidence in our work. And thus the gender difference revealed in our meta-analysis is informative.

As countries move towards higher economic level, the prevalence of obesity shift from the female to the male population [39]. However, visceral fat deposition is significantly higher in men than in women whereas subcutaneous fat accumulates more in women [40], which may be contributed by the potential role of sex hormones in instructing adipocyte metabolic programs [41]. It is thought that visceral fat deposition potentially originate from high androgen receptor density [42] and estrogen promotes the accumulation of subcutaneous fat [40]. In addition, Estrogen and estrogen receptor signaling have been found to have a protective role in liver cancer initiation and progression via the IL-6/STAT inflammatory pathways [43, 44]. Visceral fat deposition in obesity men, especially in liver, may involved in these process and contribute partially the strikingly higher male liver cancer incidence [41].

Notably, it was reported that higher free estrogen in women may promote the cell proliferation and growth in breast cancer [37, 45], kidney cancer [46, 47] and lung cancer [48, 49], but it could suppress liver cancer cell proliferation and growth [11, 50]. These discrepancy of estrogen effect may partially explain the high incidence of breast cancer, kidney cancer and lung cancer in women, but low incidence of liver cancer in women. Meanwhile, the bioavailable estradiol also increase in overweight and obese postmenopausal women, but may act differently as in the premenopausal women [45]. Unfortunately, there was no sufficient information to compare the risk of pre-menopausal women and post-menopausal women in these included studies.

Of note, overweight/obesity and its related morbidities are a growing health problem claiming 2.8 million lives annually [51], the obesity may partially contribute to the development of liver cancer. Obesity is an avoidable factor, as is smoking and alcohol. Our study suggested that an effective intervention to reduce BMI will reduce liver cancer risks, particularly in non-Asian men. On the other hand, the majority of the burden of liver cancer is in developing countries, where almost 80% of the cases are associated with chronic hepatitis B virus (HBV) or hepatitis C virus (HCV) infections, instead of obesity [52], which may be helpful to explain why the BMI–liver cancer association is higher in non-Asian than in Asian populations.

There were several potential limitations in our study that should be considered. First, although the studies had been adjusted for important risk factors, unmeasured factors related to BMI may still have influenced results of individual studies. Second, the relatively small number of studies limited conclusions from further subgroup analyses. Third, some important confounders had not been measured with sufficient precision. Only some article had considered alcohol consumption, cigarette smoking, hepatitis infection status, dietary factors and physical activity. Lack of adjustment for these important risk factors limited the ability to generalize between obesity and liver cancer.

In conclusion, the association of BMI with risk of liver cancer varies by gender. The obese men had significant higher liver cancer incidence than obese women, especially in non-Asian countries. Even stricter body weight control is strongly suggested for liver cancer prevention, especially for men in non-Asian countries.

## MATERIALS AND METHODS

### Search strategy

We searched English-language databases (PUBMED and EMBASE) and Chinese literature databases (CNKI and WanFang) to January 12, 2017 for studies on the relationship between BMI and liver cancer risk. Our research consisted of terms related to ‘body mass index’, ‘BMI’, or ‘obesity’ or ‘excess body weight’ or ‘overweight’; ‘liver cancer’ or ‘hepatocellular carcinoma’ or ‘HCC’; ‘risk’ or ‘incidence’ to identify eligible studies. No language limits were set. In addition, the reference lists of selected research papers were manually reviewed to find additional articles.

### Study selection

Studies were included in the meta-analysis if they fulfilled the following criteria: (1) published as an original article; (2) Cohort or case–control studies study in which liver cancer incidence or mortality was an outcome; (3) having clear description of normal weight, overweight and obesity defined by BMI; (4) the studies reporting risk estimates with the corresponding 95% confidence intervals (95% CI) or sufficient information to calculate them; (5) the RR and corresponding 95% CI were at least adjusted for age; (6) reported outcomes of men and women separately. When the same population was shared by multiple studies, only the study with most detailed information or the largest sample size was included.

### Data extraction

Three authors (KFY, ZML and LSW) separately screened the title and abstract of the retrieved studies and then reviewed the full texts to extract studies that met the inclusion criteria of this meta-analysis (Figure 1). NO study was excluded because the author of the study was unable to contact the published researcher. Data extraction in this meta-analysis recorded the following elements: name of the first author, publication year, study location, study type, age, follow-up years, sample size of gender, BMI measure method, BMI categories and risk estimate for each BMI category and adjustment factors. OR was used in these case-control studies and RR was used in these cohort studies. The conversion OR to RR is potentially helpful in meta-analyses, where different metrics can prevent studies from being combined [53]. The effect measure of choice for cohort studies was risk ratio (RR) and that of case-control studies was odds ratio (OR). When more than one RR was provided in a study, all of them were extracted and applied the data according to subgroup analysis.

### Quality assessment

The Newcastle-Ottawa Scale (NOS) procedure was used to assess the quality of the study. Three parameters of quality including selection, comparability, and outcome (cohort studies) or exposure (case-control studies) were included [54–56]. The NOS awards a maximum of nine points to each case- control study: four for the quality of selection (adequate case definition, representativeness of cases, selection of controls, definition of controls), two for comparability (confounding) and three for the quality of the exposure (ascertainment of exposure, same method of ascertainment of cases and controls). It awards a maximum of nine points to each cohort study: four for the quality of selection (representativeness, selection of non-exposed cohort, ascertainment of exposure, no disease at start of study), two for comparability (confounding) and three for the quality of the outcome (assessment of outcome, length of follow-up and adequacy of follow-up). Studies with NOS values of six or greater were considered moderate to high-quality studies and those with a NOS value of less than six were regarded low-quality studies.

A high-quality study was defined as a study with ≥ 7 points [55].

### Statistical analysis

We followed the WHO international classification and defined body mass categories as follows: underweight (< 18.5 kg/m^2^), normal weight (18.5 to 24.9 kg/m^2^), overweight (25.0 to 29.9 kg/m^2^) and obesity (≥ 30.0 kg/m^2^) were applied on defining BMI-categories primarily. When non-standard BMI categories were provided, the category closing to the WHO definition was selected. Data were analyzed using a random-effects model [55, 57]. Dose-response meta-analyses were conducted by using the GLST command, of which the generalized least-squares method was used for trend estimation of summarized dose-response data, based on the Greenland and Longnecker method [58]. Influence analysis was performed to estimate the influence of each individual study on the summary results Evidence of publication bias was assessed by visual inspection of funnel plots using Egger's regression test [59]. Interactions in subgroup were evaluated by random-effects analysis. A 2-tailed *P* value of < 0.05 was considered a criterion for statistical significance [60], Power calculations were performed post hoc as per the method described by Cafri et al. [61]. We based on the previous meta-analysis [62, 63] and cafri's methodology to analysis the statistical power of relative risk of the liver cancer. The macro and SAS code used were included in the online, http://link.springer.com/article/10.3758/BRM.41.1.35 Statistical analyses were performed by Stata 12.0 (Stata Corporation, College Station, TX, USA) and *P* values of two-sided less than 0.05 were considered statistically significant. Heterogeneity among studies was assessed using I^2^ statistics, which test total variation across studies [55, 56, 64, 65].

### Trial sequential analysis

In meta-analyses, it is important to minimize the risk of reaching a false-positive or false-negative conclusion. However, repeated significance tests of sparse and accumulated data are prone to yielding random errors, which increase the risk of type I errors [66]. In order to determine whether the evidence from a meta-analysis is reliable and conclusive, TSA should be used. This method assesses the risk of random errors and helps determine whether there is a need for additional trials [67]. We calculated the required information size based on a relative risk reduction of 15 % in incidence and mortality of liver cancer. The type I error (α) and power (1 – β) were set as 0.05 and 0.80, respectively. TSA was conducted using stata12.0. The blue line shows the cumulative Z-score of the meta-analysis, and the inward sloping red dash lines represent the truncated trial sequential monitoring boundaries.

## SUPPLEMENTARY MATERIALS FIGURES AND TABLE


