# Supplementary material for: Meta-analysis reveals gender difference in the association of liver cancer incidence and excess BMI
Source: Oncotarget. 2017 Aug 10;8(42):72959–71. doi: 10.18632/oncotarget.20127 (PMC5641183; doi:10.18632/oncotarget.20127)
Supplement: Supplementary file 1 [file oncotarget-08-72959-s001.pdf]

Meta-analysis reveals gender difference in the association of liver cancer incidence and excess BMI

SUPPLEMENTARY MATERIALS

A

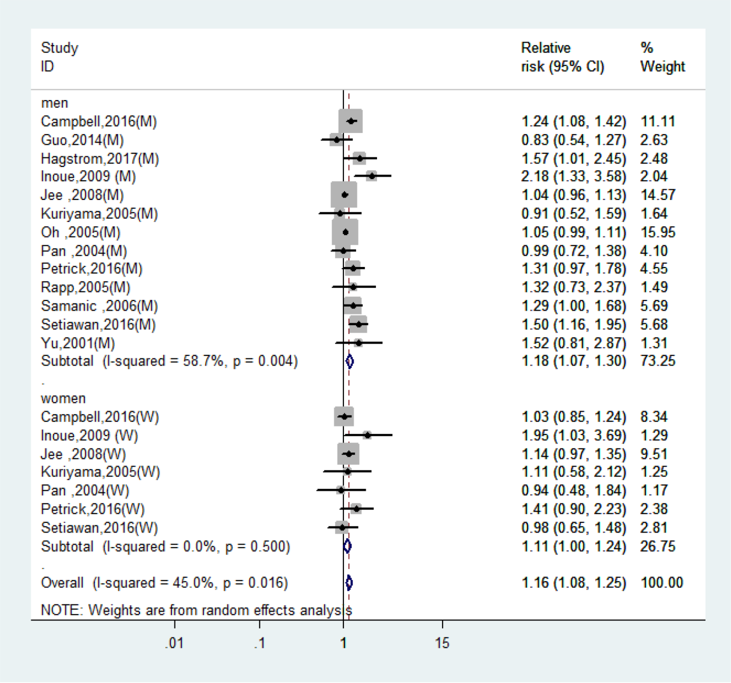

B

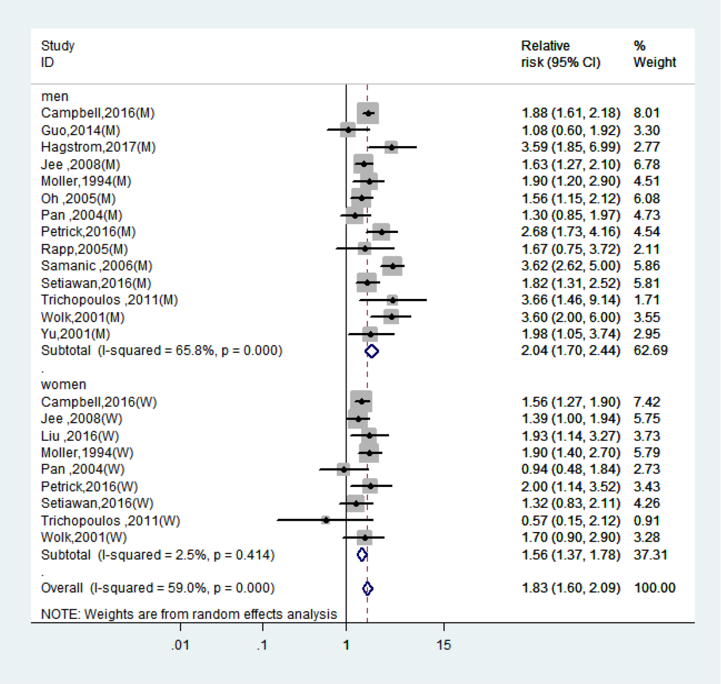

Supplementary Figure 1: The relative risks of liver cancer incidence in overweight and obesity. (**A**) Forest plots of the overweight vs. normal weight; (**B**) Forest plots of the obesity vs. normal weight. CI, confidence intervals; W, women; M, men.

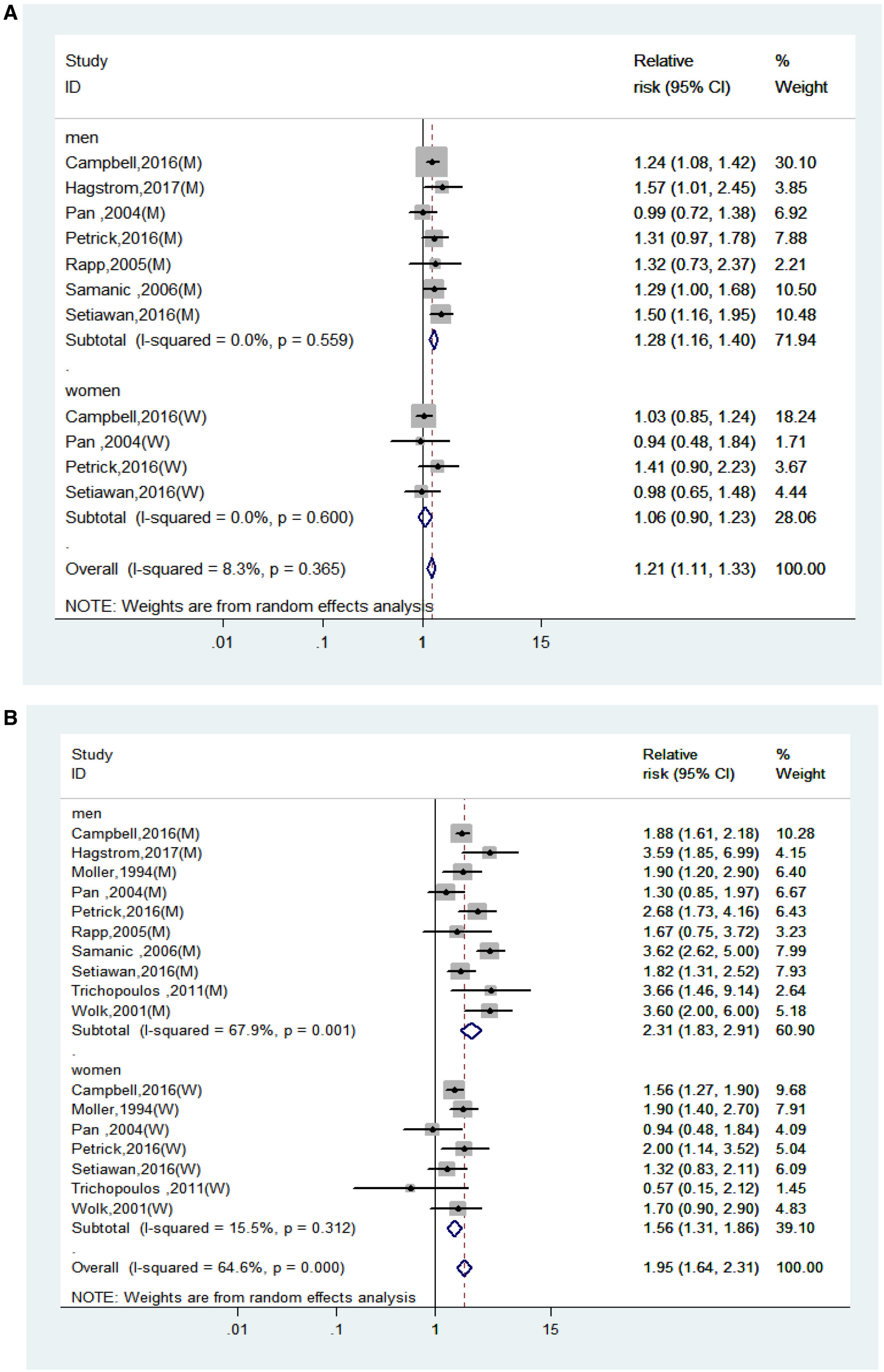

Supplementary Figure 2: The relative risks of non-Asian overweight and obesity. (**A**) Forest plots of the non-Asian overweight vs. normal weight; (**B**) Forest plots of the non-Asian obesity vs. normal weight. CI, confidence intervals; W, women; M, men.

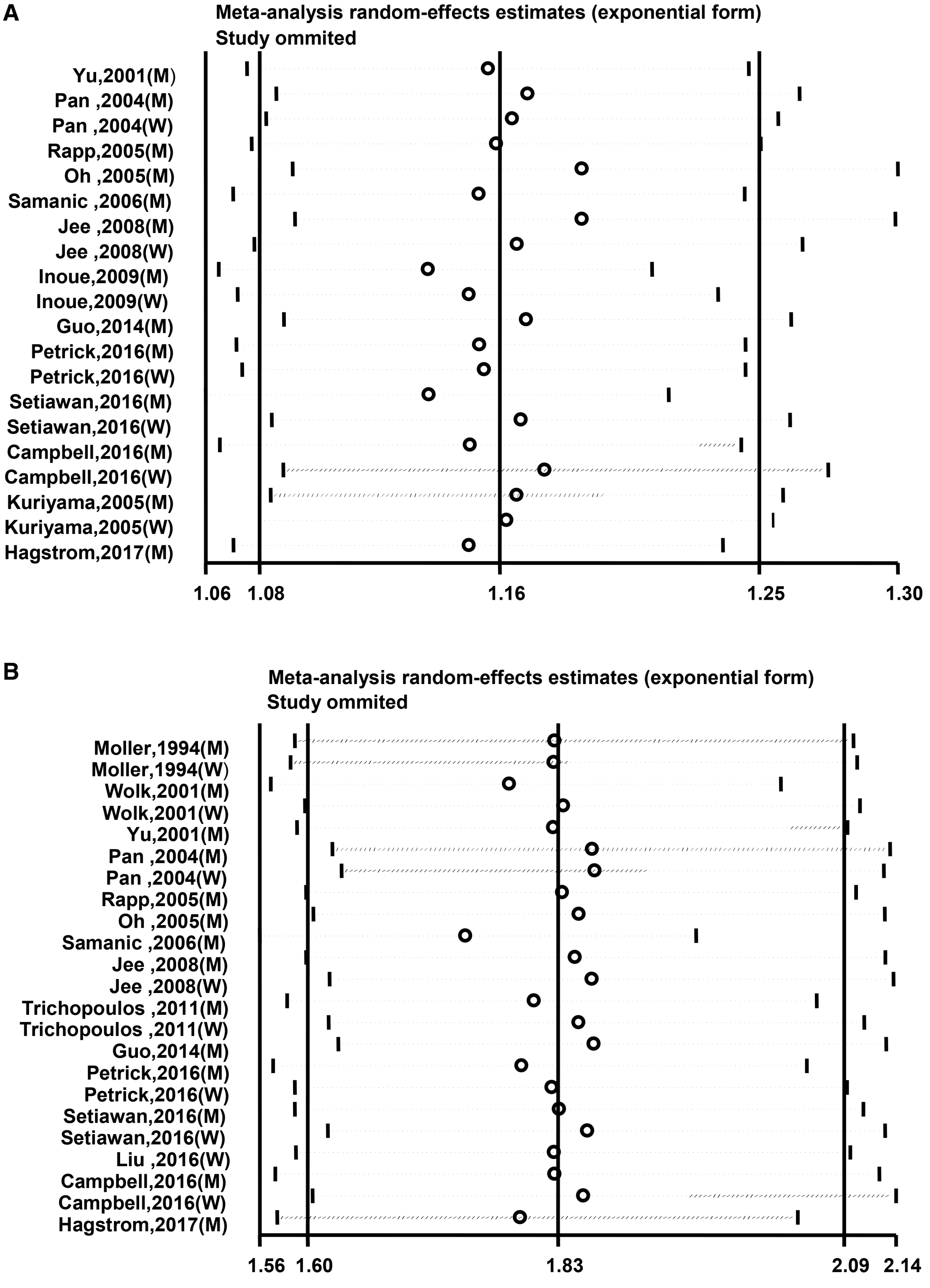

Supplementary Figure 3: Sensitivity analysis of the studies included in overall overweight (**A**) and obesity (**B**). W, women; M, men.

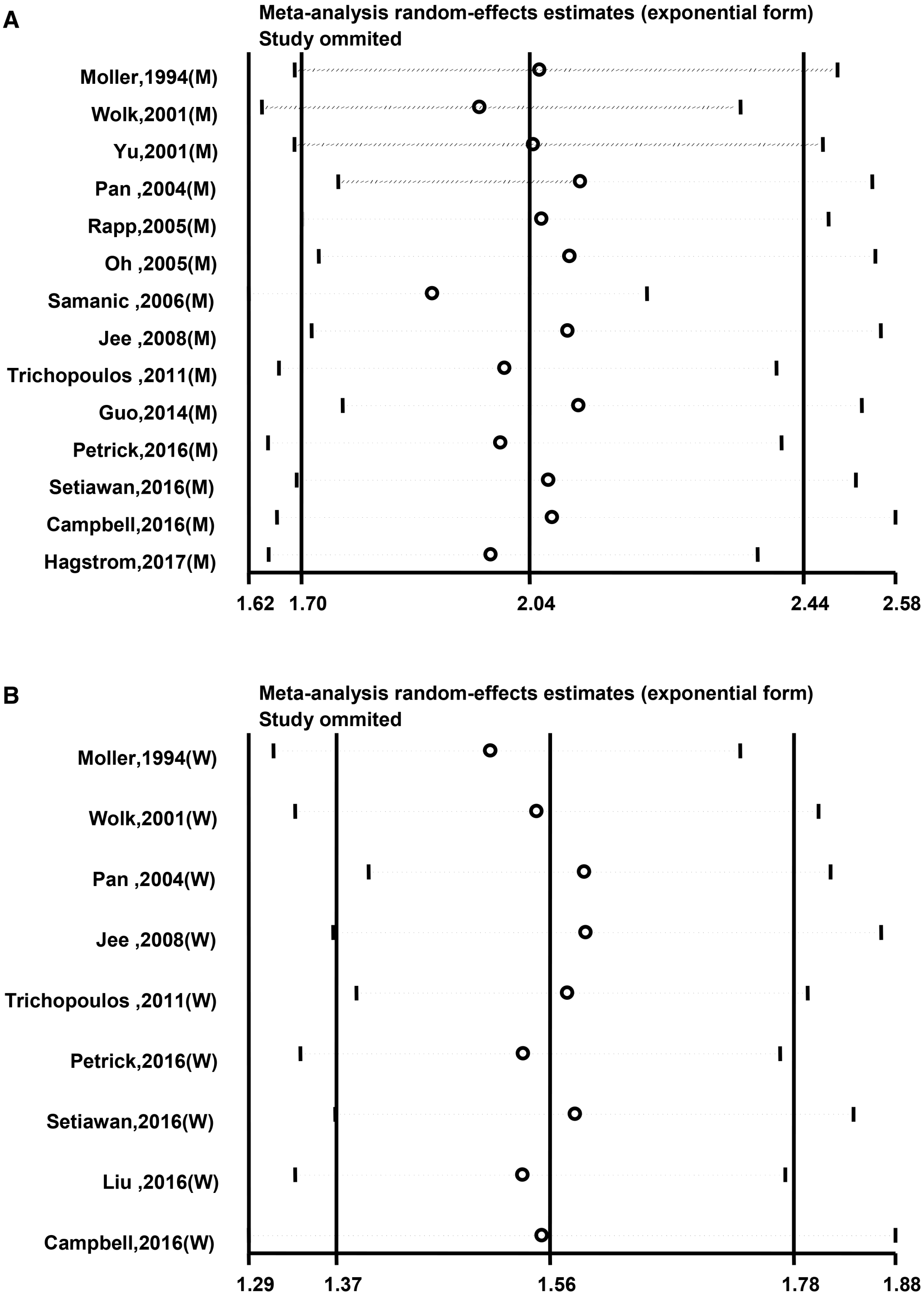

Supplementary Figure 4: Sensitivity analysis of the studies included in overall obesity men (**A**) and women (**B**). W, women; M, men.

A

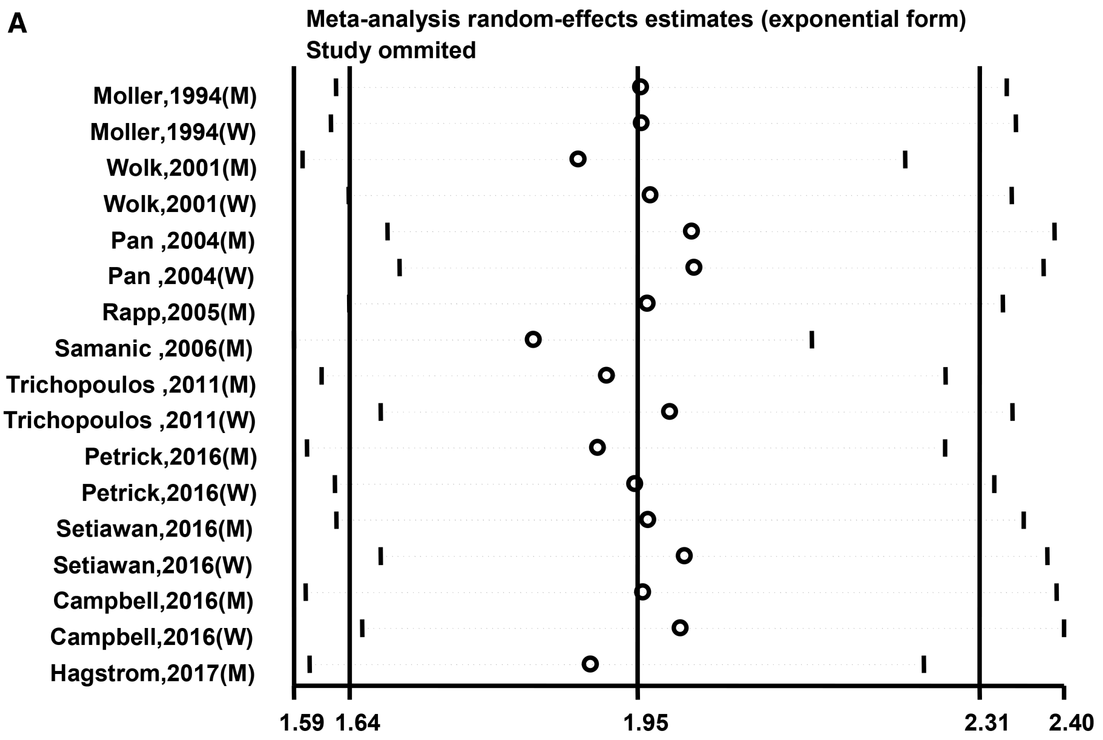
Meta-analysis random-effects estimates (exponential form)
Study ommited
Moller,1994(M)
Moller,1994(W)
Wolk,2001(M)
Wolk,2001(W)
Pan ,2004(M)
Pan ,2004(W)
Rapp,2005(M)
Samanic ,2006(M)
Trichopoulos ,2011(M)
Trichopoulos ,2011(W)
Petrick,2016(M)
Petrick,2016(W)
Setiawan,2016(M)
Setiawan,2016(W)
Campbell,2016(M)
Campbell,2016(W)
Hagstrom,2017(M)
1.59
1.64
1.95
2.31
2.40

B

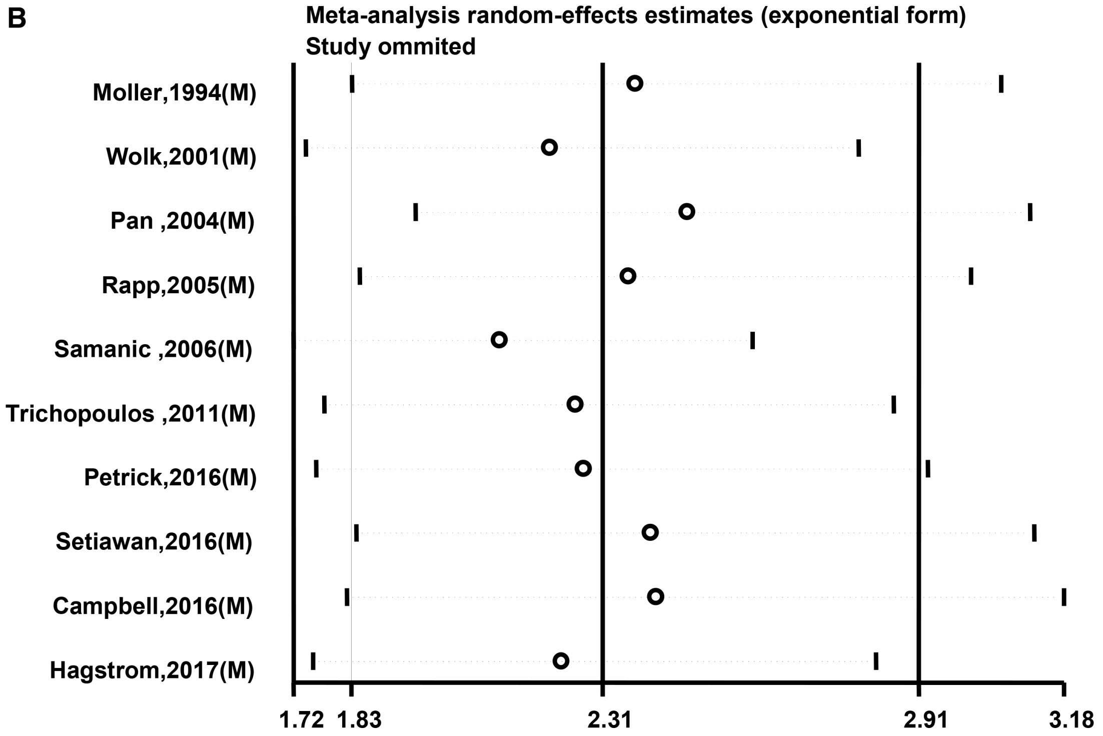
Meta-analysis random-effects estimates (exponential form)
Study ommited
Moller,1994(M)
Wolk,2001(M)
Pan ,2004(M)
Rapp,2005(M)
Samanic ,2006(M)
Trichopoulos ,2011(M)
Petrick,2016(M)
Setiawan,2016(M)
Campbell,2016(M)
Hagstrom,2017(M)
1.72
1.83
2.31
2.91
3.18

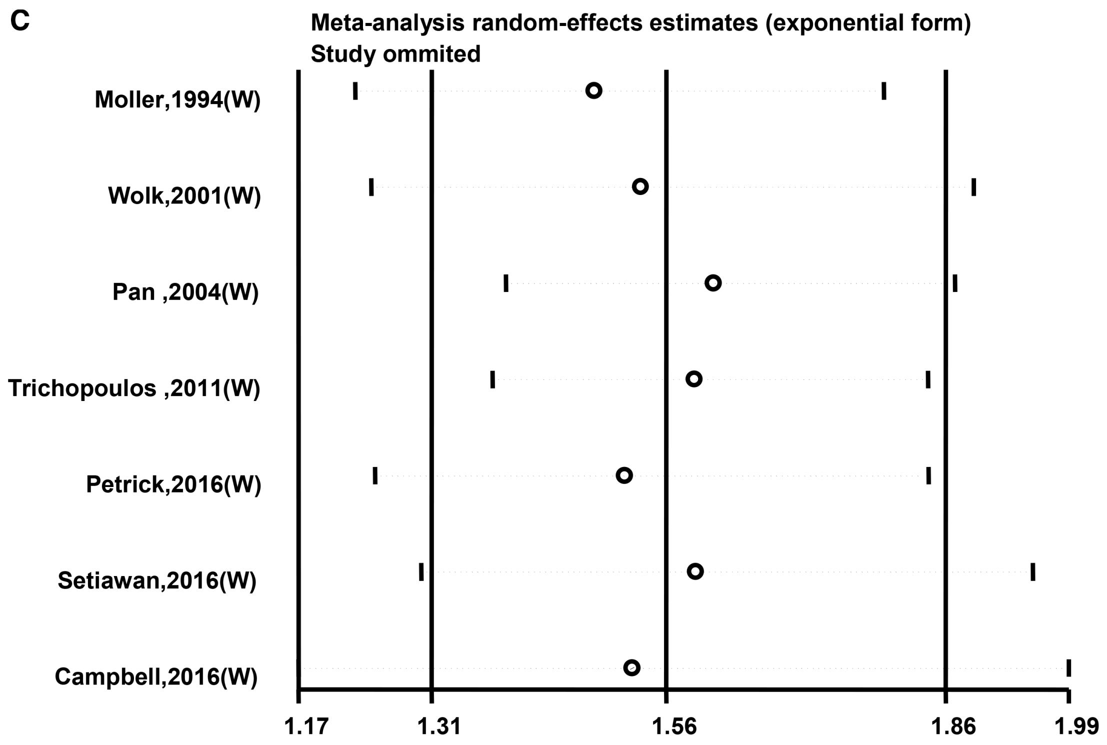

Supplementary Figure 5: Sensitivity analysis of the studies included in non-Asian overall obesity (**A**), men (**B**) and women (**C**). W, women; M, men.

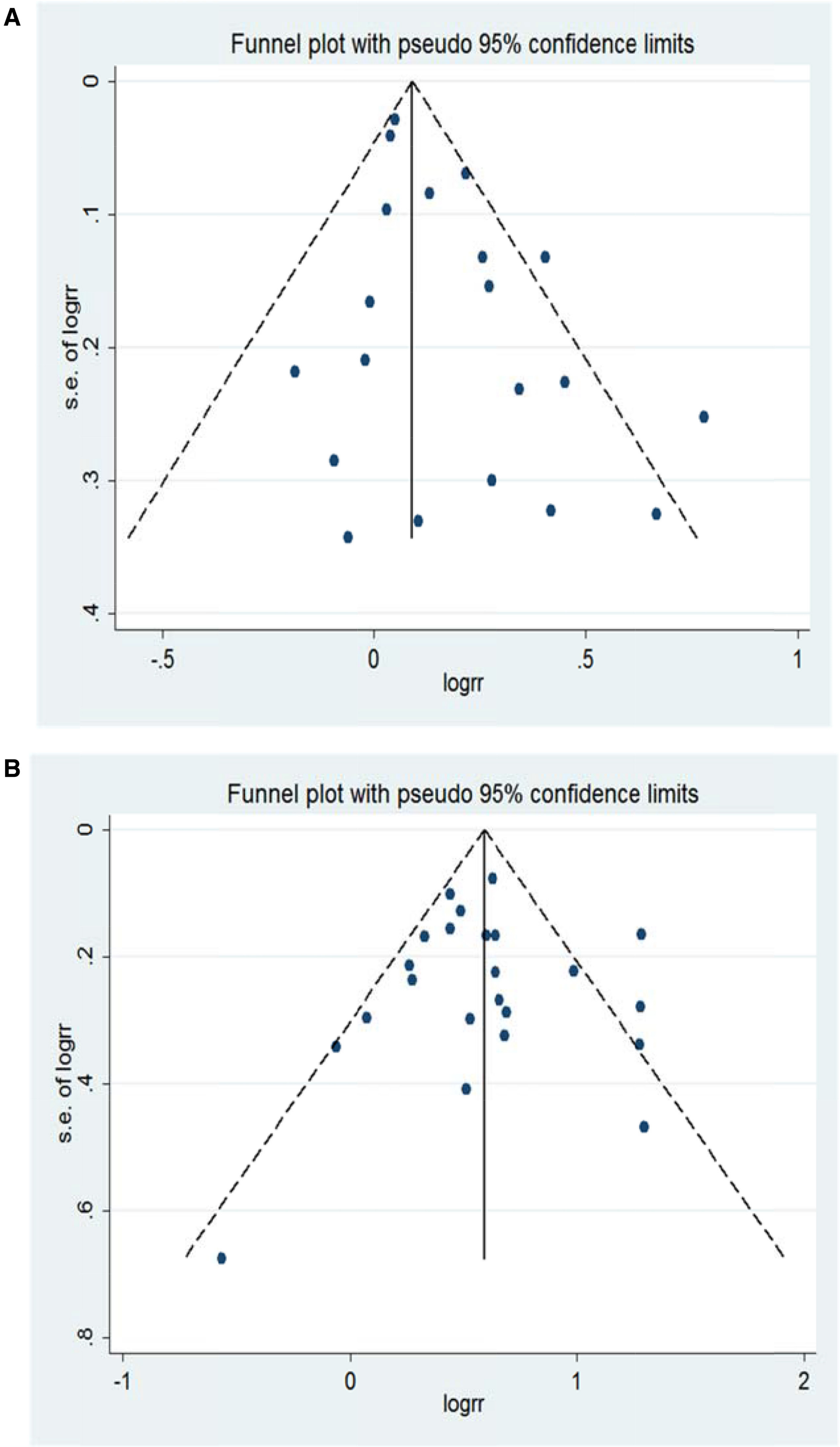

Supplementary Figure 6: Funnel plot for all studies included in the meta-analysis of BMI and liver cancer incidence. (**A**) overweight study (p = 0.022 by Egger's test); (**B**) obesity study (p = 0.900 by Egger's test).

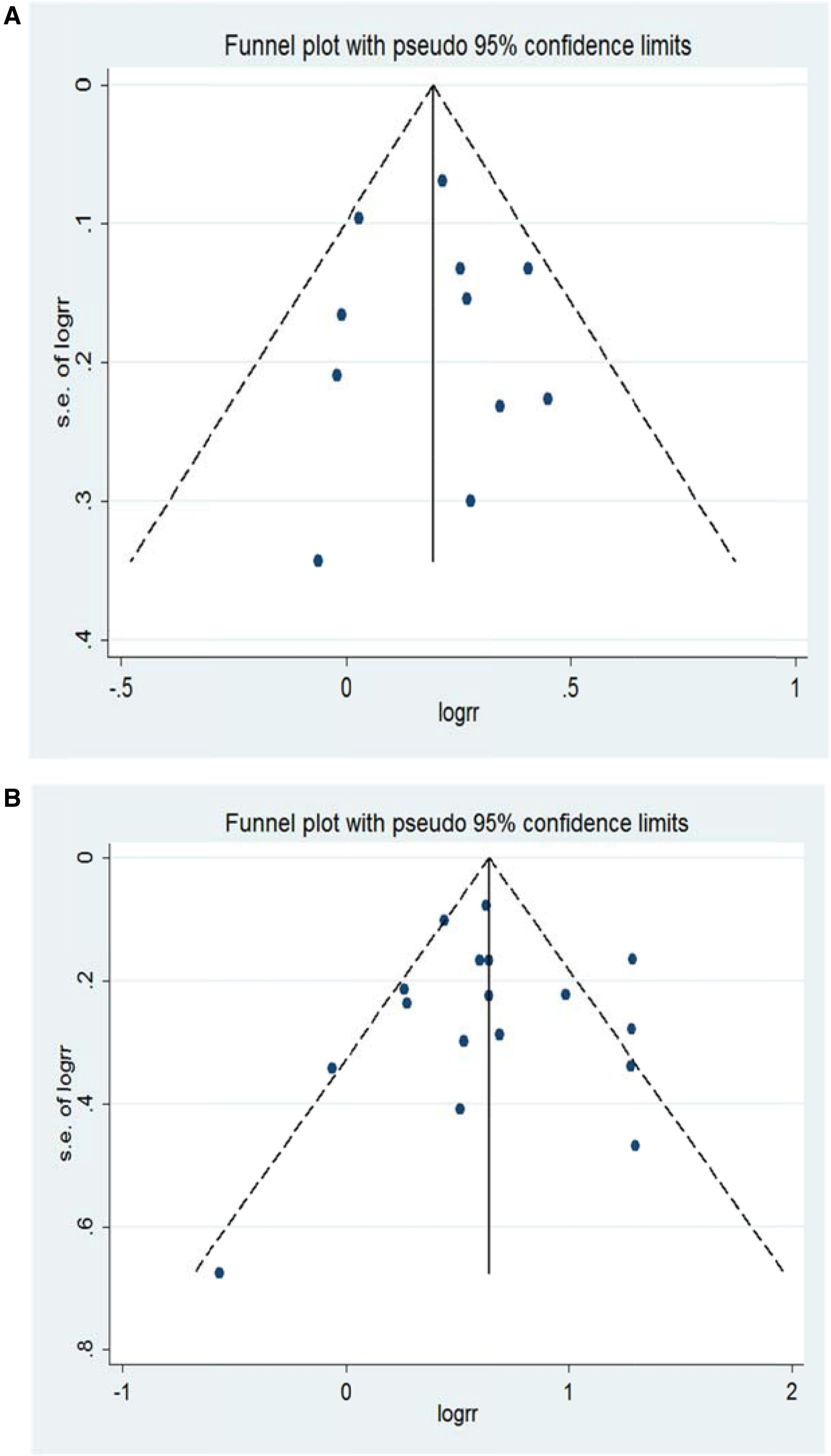

Supplementary Figure 7: Funnel plot for non-Asian studies included in the meta-analysis. (**A**) overweight, $p = 0.877$ by Egger's test; (**B**) obesity, $p = 0.794$ by Egger's test.

A

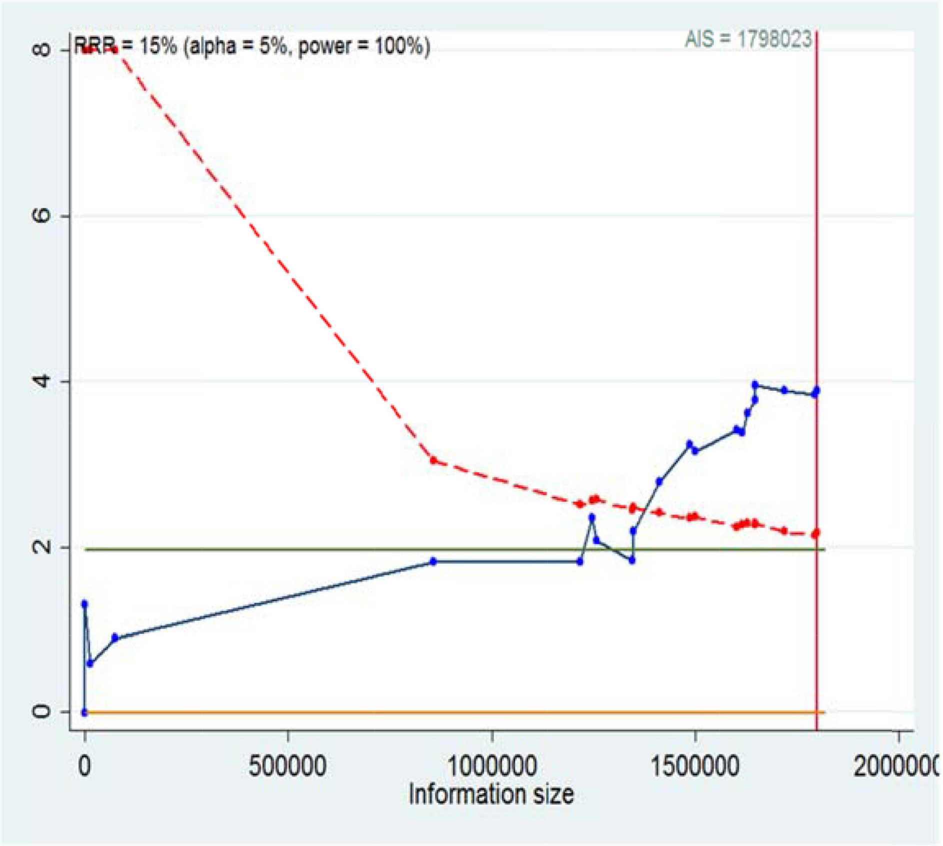

B

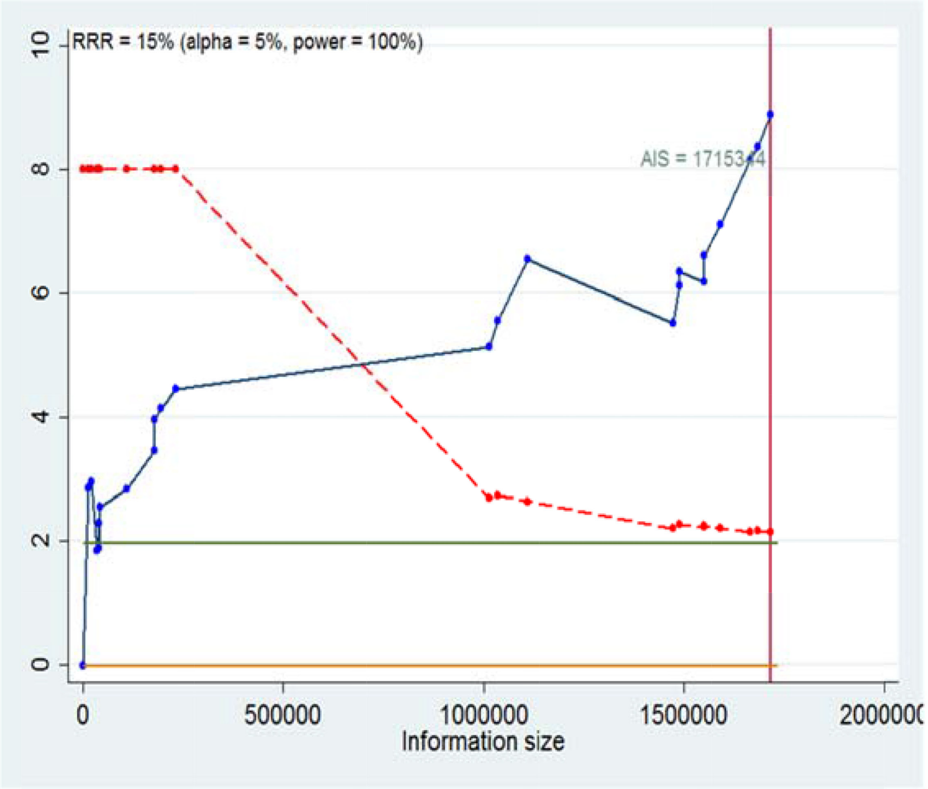

Supplementary Figure 8: Trial sequential analysis for the study of overall overweight (**A**) or obesity (**B**) and liver cancer incidence. (A) the AIS = 1798023, α = 0.05, power = 100%; (B) Trial sequential analysis of the obesity. b, the AIS = 1715144, α = 0.05, power = 100%. AIS: accrued information size.

A

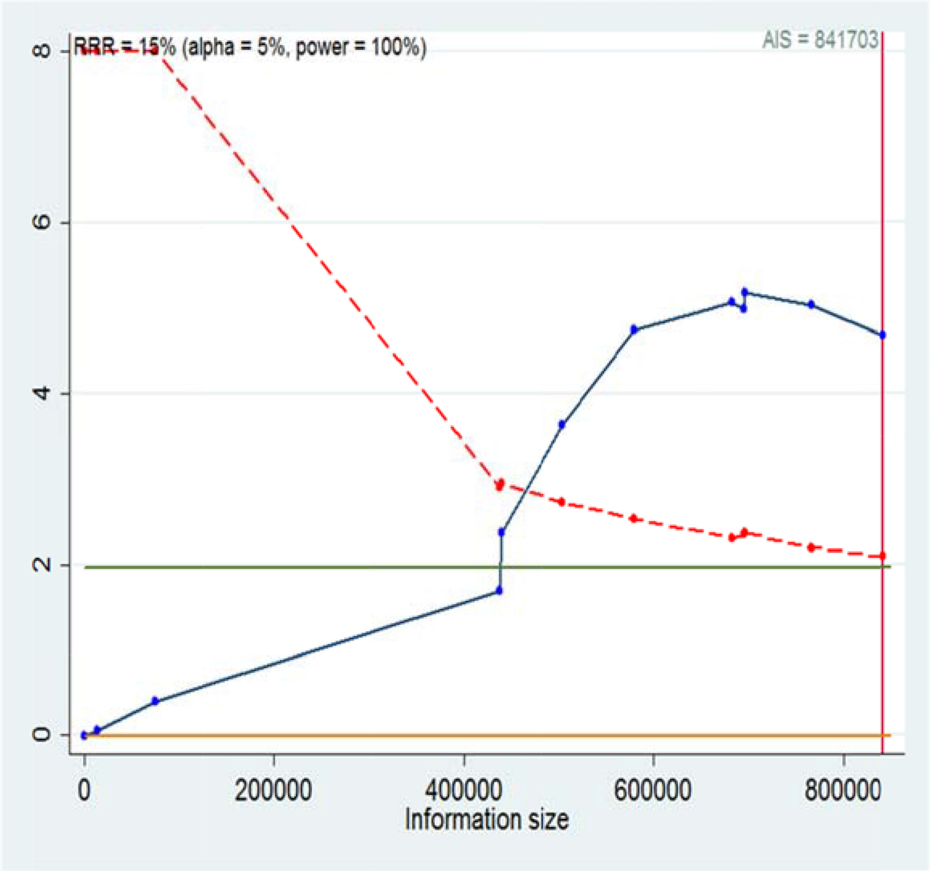

B

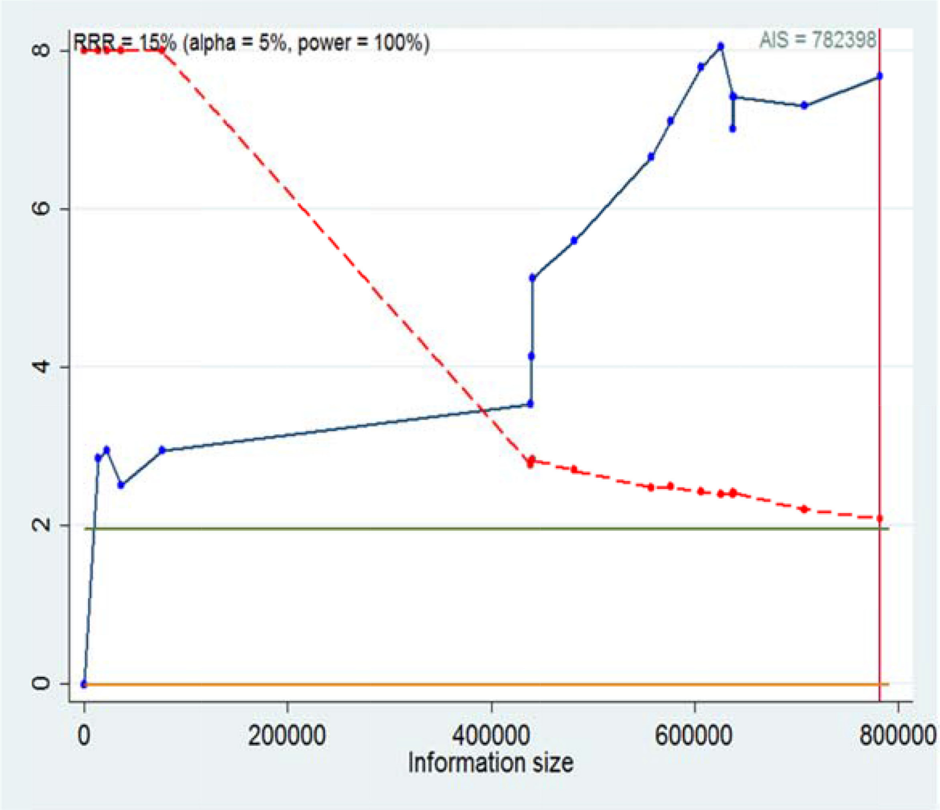

Supplementary Figure 9: Trial sequential analysis for the study of non-Asian overweight (**A**) or obesity (**B**). a, the AIS = 841703, α = 0.05, power = 100%. b, the AIS = 782398, α = 0.05, power = 100%. AIS: accrued information size.

Supplementary Table 1: Assessment of study quality

Studies (all cohort studies)	Quality Indicators From Newcastle-Ottawa Scale								
	1	2	3	4	5A	5B	6	7	8
Campbell et al. 2016	☆	☆		☆	☆	☆	☆	☆	☆
Guo et al. 2014	☆	☆	☆	☆	☆	☆	☆		☆
Hagstrom, 2017	☆	☆	☆	☆			☆	☆	☆
Inoue et al. 2009,	☆	☆	☆	☆	☆	☆	☆	☆	☆
Jee et al. 2008	☆	☆	☆	☆		☆	☆	☆	☆
Kuriyama et al. 2005	☆	☆		☆	☆	☆	☆		☆
Liu et al. 2016	☆	☆	☆	☆	☆	☆	☆	☆	☆
Moller et al. 1994	☆	☆	☆	☆			☆	☆	☆
Oh et al. 2005	☆	☆	☆	☆		☆		☆	☆
Pan et al. 2004		☆	☆	☆	☆	☆	☆	☆	☆
Petrick et al. 2016	☆	☆	☆	☆		☆		☆	☆
Rapp et al. 2005	☆	☆	☆	☆	☆	☆	☆		☆
Samanic et al. 2006,	☆	☆	☆	☆		☆	☆	☆	☆
Setiawan et al. 2016	☆		☆	☆	☆	☆	☆	☆	☆
Trichopoulosetal, 2011	☆	☆	☆	☆	☆	☆	☆	☆	☆
Wolk et al. 2001	☆	☆	☆	☆			☆	☆	☆
Yu et al. 2001		☆	☆	☆	☆	☆	☆	☆	☆
